# T-Cell Cytokine Gene Polymorphisms and Vitamin D Pathway Gene Polymorphisms in End-Stage Renal Disease due to Type 2 Diabetes Mellitus Nephropathy: Comparisons with Health Status and Other Main Causes of End-Stage Renal Disease

**DOI:** 10.1155/2014/120317

**Published:** 2014-12-22

**Authors:** Alicja E. Grzegorzewska, Grzegorz Ostromecki, Paulina Zielińska, Adrianna Mostowska, Paweł P. Jagodziński

**Affiliations:** ^1^Department of Nephrology, Transplantology and Internal Diseases, Poznań University of Medical Sciences (PUMS), 49 Przybyszewskiego Boulevard, 60-355 Poznań, Poland; ^2^DaVita Clinic Piła Dialysis Center, Wojska Polskiego 43, 64-420 Piła, Poland; ^3^Student Nephrology Research Group, Department of Nephrology, Transplantology and Internal Diseases, PUMS, Przybyszewskiego 49, 60-355 Poznań, Poland; ^4^Department of Biochemistry and Molecular Biology, PUMS, Święcickiego 6, 60-781 Poznań, Poland

## Abstract

*Background*. T-cell cytokine gene polymorphisms and vitamin D pathway gene polymorphisms were evaluated as possibly associated with end-stage renal disease (ESRD) resulting from type 2 diabetes mellitus (DM) nephropathy. *Methods*. Studies were conducted among hemodialysis (HD) patients with ESRD due to type 2 DM nephropathy, chronic glomerulonephritis, chronic infective tubulointerstitial nephritis, and hypertensive nephropathy as well as in healthy subjects. A frequency distribution of T-cell-related interleukin (IL) genes (*IL18* rs360719, *IL12A* rs568408, *IL12B* rs3212227, *IL4R* rs1805015, *IL13* rs20541, *IL28B* rs8099917, *IL28B*, and rs12979860) and vitamin D pathway genes (GC genes: rs2298849, rs7041, and rs1155563; VDR genes: rs2228570, rs1544410; and RXRA genes: rs10776909, rs10881578, and rs749759) was compared between groups. *Results*. No significant differences in a frequency distribution of tested polymorphisms were shown between type 2 DM nephropathy patients and controls. A difference was found in *IL18* rs360719 polymorphic distribution between the former group and chronic infective tubulointerstitial nephritic patients (*P*_trend_ = 0.033), which also differed in this polymorphism from controls (*P*_trend_ = 0.005). *Conclusion*. T-cell cytokine and vitamin D pathway gene polymorphisms are not associated with ESRD due to type 2 DM nephropathy in Polish HD patients. *IL18* rs360719 is probably associated with the pathogenesis of chronic infective tubulointerstitial nephritis.

## 1. Introduction

Diabetes mellitus (DM) is the most common cause of end-stage renal disease (ESRD) in many hemodialysis (HD) centers. In Australia and New Zealand, the incident ESRD population (1991–2005) who began renal replacement therapy (RRT) included 30.0% type 2 DM and 4.5% type 1 DM subjects [1]. In the HEMODIALYSIS (HEMO) study, the group of HD patients comprised approximately 45% of DM subjects [2].

Diabetic ESRD patients compared to nondiabetic ESRD subjects show higher both mortality rate [3] and prevalence of coronary artery disease (CAD) [4], are more prone to severe infections [5] and worse response to hepatitis B vaccination [6], and more often suffer from adynamic bone disease associated with low serum parathyroid hormone (PTH) levels [7]. In this paper we will focus on ESRD due to type 2 DM nephropathy. Together with altered glucose metabolism and insulin resistance, deficiency of vitamin D [8] and aberrant T-cell cytokine balance [9] were found to be associated with this severe complication of type 2 DM. There is a link between vitamin D and T-cell functional balance: active form of vitamin D [1,25(OH)_2_D] has the inhibitory effect on the T helper (Th) 17 and Th1 response [10].

Abnormalities in T-cell cytokine equilibrium [11–13] and plasma vitamin D concentrations [14–16] are related to cardiovascular events [13, 16] and immunononcompetence during infections [11, 14] and vaccinations [12, 15]. Serum PTH levels are dependent on serum vitamin D concentrations [17], and T cells are implicated in the mechanism of PTH action in bone [18].

Vitamin D activity may be adequately expressed if vitamin D pathway components (vitamin D binding protein, also referred to as group-specific component (GC), vitamin D receptor (VDR), and retinoid X receptors (RXRs)) are properly structured and regulated. The recent study by Zhang et al. [19] has shown that* VDR *BsmI polymorphism correlates with type 2 DM nephropathy and may be susceptible for early onset of this nephropathy. Among T-cell-related cytokine gene polymorphisms, promoter polymorphic variants of* IL*10 [20, 21] and* IL6 *[22] were already associated with the risk of type 2 DM nephropathy. Monocyte chemoattractant protein 1 (MCP-1) has been reported to participate in the pathogenesis of early type 2 DM nephropathy [23], but *MCP1* polymorphism in the promoter region was not differentially distributed between ESRD patients with type 2 DM nephropathy and healthy controls [24, 25].

To our knowledge, there are scarce data, if any, on ESRD due to type 2 DM nephropathy showing a frequency distribution of single nucleotide polymorphisms (SNPs) of T-cell-related IL genes:* IL18 *rs360719,* IL12A *rs568408,* IL12B *rs3212227,* IL4R *rs1805015,* IL13 *rs20541,* IL28B *rs8099917, and* IL28B *rs12979860 as well as vitamin D pathway genes: GC genes (*GC *rs2298849, rs7041, and rs1155563), VDR genes (*VDR *rs2228570, rs1544410), and RXR *α* genes (*RXRA *rs10776909, rs10881578, and rs749759). The aim of our study was to determine the potential association between aforementioned polymorphisms of T-cell-related cytokine genes and vitamin D pathway genes and ESRD due to type 2 DM nephropathy. For comparisons, aforementioned genotype frequencies of healthy controls as well as ESRD patients with other main causes of ESRD were used. Polymorphism related associations, if exist, could contribute to explanation of susceptibility to ESRD due to type 2 DM nephropathy and phenotype differences between ESRD patients with type 2 DM nephropathy and other causes of ESRD.

## 2. Material and Methods

### 2.1. Patients and Controls

Blood samples for genotype analyses are collected since 2009 from ESRD patients (estimated glomerular filtration rate (eGFR) category G5 in accordance with KDIGO recommendations [26]). All subjects were treated with HD on enrolment. Controls were recruited from blood donors and healthy volunteers unrelated to patients. All enrolled individuals live/lived in the Greater Poland region of Poland.

Genotyping of* IL18 *rs360719,* IL12A *rs568408,* IL12B *rs3212227,* IL4R *rs1805015, and* IL13 *rs20541 polymorphisms was performed in 2009–2012 using currently available material. Results had been analyzed in our previous studies in the context of responsiveness to the surface antigen of hepatitis B virus (HBsAg) using data of all (not segregated) patients [27–30]. For this study, we used results of controls and patients with type 2 DM nephropathy, chronic glomerulonephritis, chronic infective tubulointerstitial nephritis, and hypertensive nephropathy.

*IL28B *rs8099917,* IL28B *rs12979860,* GC *rs2298849,* GC *rs7041,* GC *rs1155563,* VDR *rs2228570,* VDR *rs1544410,* RXRA *rs10776909,* RXRA *rs10881578, and* RXRA *rs749759 polymorphisms were analyzed in winter 2013/2014 among HD patients with ESRD (*n* = 893) due to type 2 DM nephropathy (*n* = 366), chronic glomerulonephritis (*n* = 178), chronic infective tubulointerstitial nephritis (*n* = 118), and hypertensive nephropathy (*n* = 231) as well as healthy controls (*n* = 378).

DM was not diagnosed in patients having renal diseases other than type 2 DM nephropathy.

Healthy individuals and HD patients with other renal diseases as cause of ESRD served as reference groups for a frequency distribution of tested polymorphic variants. All examined subjects were of Caucasian race.

Basic clinical and laboratory data were collected on enrolment and they are updated every year.

### 2.2. Genotyping

Genomic DNA for genotype analysis was isolated from peripheral blood lymphocytes by salt-out extraction procedure.

Genotyping of* IL18 *rs360719,* IL12A *rs568408,* IL12B *rs3212227,* IL4R *rs1805015, and* IL13 *rs20541 polymorphisms was performed as previously described [27–30].

*IL28B *rs8099917 and* IL28B *rs12979860 polymorphisms were genotyped using high-resolution melting curve analysis (HRM) on the LightCycler 480 system (Roche Diagnostics, Mannheim, Germany) with the use of 5x HOT FIREPol EvaGreen HRM Mix (Solis BioDyne, Tartu, Estonia). The PCR program consisted of an initial step at 95°C for 15 min to activate HOT FIREPol DNA polymerase, followed by 50 amplification cycles of denaturation at 95°C for 10 s, annealing at 61°C for 10 s, and elongation at 72°C for 15 s. Amplified DNA fragments were then subjected to HRM with 0.1°C increments in temperatures ranging from 76 to 96°C. Primers used for PCR with subsequent HRM analysis were as follows: rs8099917F 5′TTTGTCACTGTTCCTCCTTTTG3′, rs8099917R 5′AAGACATAAAAAGCCAGCTACCA3′, rs12979860F 5′CGTGCCTGTCGTGTACTGAA3′, and rs12979860R 5′AGGCTCAGGGTCAATCACAG3′.

Genotyping of the* GC *rs1155563,* GC* rs2298849,* RXRA *rs10881578, and* RXRA *rs10776909 polymorphisms was carried out by HRM on the Bio-Rad CFX96 Real Time PCR system (Bio-Rad, Hercules, CA). DNA fragments amplified with the use of specific primers were subjected to HRM with 0.1°C increments in temperatures ranging from 71 to 92°C. Genotyping of the* GC *rs7041,* RXRA* rs749759,* VDR *rs1544410, and* VDR *rs2228570 was performed using the polymerase chain reaction and restriction fragment length polymorphism (PCR-RFLP) method according to the manufacturer's instructions (Fermentas, Vilnius, Lithuania). Primer sequences and conditions for HRM and PCR-RFLP analyses are presented in Table 1.

For quality control, the genotyping analysis was blinded to the subject's case-control status. In addition, approximately 10% of the randomly chosen samples were regenotyped. Samples that failed the genotyping were excluded from further statistical analyses.

### 2.3. 25(OH)D Testing

Plasma 25(OH)D was determined in blindly selected 162 HD patients in the winter season of the year to avoid differences in sunlight exposure between patients who used to sunbathe and those who did not. Plasma 25(OH)D concentration was measured in HD patients who had not been treated with vitamin D or had stopped such a treatment for at least 3 weeks to obtain the so-called basic vitamin D concentrations. Under these conditions, there were no patients showing optimal plasma 25(OH)D levels (35–80 ng/mL for adults). To examine plasma 25(OH)D levels, a chemiluminescent microparticle immunoassay (CMIA) was used according to the manufacturer's instructions (Abbott Diagnostics ARCHITECT 25-OH VITAMIN D CMIA).

### 2.4. Statistical Methods

Results are presented as percentage for categorical variables, as mean with one standard deviation for normally distributed continuous variables or as median with range for not normally distributed continuous variables as tested by the Shapiro-Wilk test. Statistical tests used for comparison of data obtained in selected groups are indicated at *P* values.

Hardy-Weinberg equilibrium (HWE) was tested to compare the observed genotype frequencies to the expected ones using Chi-square test. Distributions of tested polymorphisms were consistent with HWE with three exceptions which are indicated in tables showing analysis of genotype and allele distributions. The Fisher exact probability test or Chi-square test was used to evaluate differences in genotype and allele prevalence between the examined groups. Homozygotes for the major allele were the reference group. The odds ratio (OR) with *P* value and 95% confidence intervals (95% CI) value were calculated. All probabilities were two-tailed. Polymorphisms were tested for association using the Chi-square test for trend (*P*_trend_). Power analysis was performed by Fisher's exact test.

Values of *P* < 0.05 were judged to be significant. However, associations were reported only if the following conditions were fulfilled.

1. A genotype distribution was consistent with HWE in a tested group and a referent group.
2. *P*_trend_ was below 0.05.
3. Odds ratio remained significant after the Bonferroni correction applied for multiple testing, if appropriate.

Aforementioned statistical calculations were performed using GraphPad InStat 3.10, 32 bit for Windows, created on July 9, 2009 (GraphPad Software, Inc., La Jolla, USA), CytelStudio version 10.0, created on January 16, 2013 (CytelStudio Software Corporation, Cambridge, USA), and Statistica version 10, 2011 (StatSoft, Inc., Tulsa, USA).

## 3. Results

Characteristics of the examined HD patients are presented in Tables 2 and 3. ESRD patients due to type 2 DM nephropathy compared to non-DM ESRD patients showed older age at RRT onset, shorter treatment with RRT, higher death rate on RRT, higher prevalence of CAD and myocardial infarction, lower serum PTH level, and lower frequency of parathyroidectomy and treatment with cinacalcet.

In respect of the examined parameters, type 2 DM nephropathy patients differed the most significantly from chronic glomerulonephritic subjects, the least significantly from hypertensive nephropathy patients.

There were no differences in frequency distributions of tested genotypes between type 2 DM nephropathy patients and healthy subjects (Table 4) as well as other ESRD patients analyzed together (Table 5) which could be judged as significant associations.

Comparisons of genotype and allele frequencies between type 2 DM nephropathy patients and other ESRD groups revealed associations only with chronic infective tubulointerstitial nephritic patients in respect of* IL18 *rs360719 (Table 6, no significant results are shown). Frequency of* IL18 *rs360719 allele C carriers was higher in type 2 DM nephropathy patients than in those with chronic infective tubulointerstitial nephritis. The latter group showed lower frequency of* IL18 *rs360719 allele C carriers compared to healthy controls (Table 6).

Type 2 DM nephropathy patients with diagnosed CAD differed in tested genotype frequencies neither from type 2 DM nephropathy subjects without CAD (Table 7) nor from healthy controls (Table 8).

## 4. Discussion

Genetic studies involving DM nephropathy and related complications are not consistent in many aspects [31–34]. Some polymorphisms tested in this study were reported as being associated with type 1 DM (*IL12B *rs3212227 [35],* IL4R *[36, 37],* IL13 *[37],* VDR* rs1544410 [38, 39], and* VDR *rs2228570 [38]), type 2 DM susceptibility (*VDR *rs2228570 [40],* VDR* rs1544410 [41]), and phenotype of type 2 DM (*VDR *rs2228570 [42],* VDR* rs1544410 [41, 43]).* VDR *rs2228570 and* IL4 *polymorphisms were also related to the risk of chronic kidney disease [44, 45]. On the other hand, there are also data indicating no major effect of* IL12B *on type 1 DM susceptibility in the entire study group [46], no association of* IL4R *with type 1 DM [47], no evident causal relationship between vitamin D pathway genes and type 2 DM, myocardial infarction or mortality [48], similar distribution of genotypes, allele and haplotypes of* VDR *rs2228570 and* VDR* rs731236 between type 2 DM patients and controls [49], no contribution of* VDR* rs1544410 to type 1 DM susceptibility [50], and no association of* VDR* rs1544410 with chronic kidney disease susceptibility [51].

In this study we were not able to show significant differences in the frequency distribution of tested polymorphic variants of T-cell-related cytokine genes or vitamin D pathway genes between HD patients with ESRD due to type 2 DM nephropathy and controls as well as HD patients with other causes of ESRD analyzed together. This lack of association was present although the examined type 2 DM nephropathy patients showed clinical complications more frequently than HD patients with other renal diseases: higher dialysis related mortality rate [3], higher prevalence of CAD including myocardial infarction [4], lower serum PTH, and lower frequency of parathyroidectomy and treatment with cinacalcet, all of them predictive for higher tendency to adynamic bone disease [7]. Type 2 DM nephropathy patients with or without diagnosis of CAD also did not differ in tested genotype distributions.

Development of ESRD substantially ameliorates interpatient clinical variability related to underlying renal impairment and exposes uremia-related signs and symptoms. Comparisons of type 2 DM nephropathy patients in respect of tested genotype frequencies with subjects showing other common causes of ESRD revealed that the former group has a higher* IL18 *rs360719 minor allele frequency than chronic infective tubulointerstitial nephritic group. In this case, lower* IL18 *rs360719 minor allele frequency in tubulointerstitial nephritic patients was observed also when their results were compared to those of healthy subjects. Sánchez et al. [52] have found a significant increase in the relative expression of IL-18 mRNA in individuals carrying the rs360719 minor allele. IL-18 is IFN-*γ* inducing factor. Infective tubulointerstitial nephritic patients are known to have diminished ability of blood leukocytes to produce IFN-*γ* [53]. Our study indicates that this may be related to lower frequency of* IL18 *rs360719 minor allele in this group compared to controls and type 2 DM nephropathy patients. In type 2 DM patients with overt nephropathy, positive correlations between plasma IFN-*γ*, proteinuria, and eGFR were found [54].

Due to limited financial support, we did not perform any functional studies regarding T-cell-related interleukin and vitamin D pathway genes, especially that multiple influences independent or dependent on genetic profile need to be taken into account in such studies conducted in the uremic milieu. Although the examined patients showing ESRD due to type 2 DM nephropathy were well-defined group, they obviously were not consistent in HLA DRB1 alleles. The latter could be important in modulating susceptibility to advanced type 2 DM nephropathy and related complications, like it was shown for type 1 DM [55] or type 2 DM [41], regardless of their complications.

## 5. Summary 

Distributions of tested T-cell cytokine gene polymorphisms or vitamin D pathway gene polymorphisms are not significantly different among patients with ESRD due to type 2 DM nephropathy and healthy individuals. Subjects with ESRD due to type 2 DM nephropathy differ in clinical manifestation from patients with other nephropathies leading to dialysis dependency, but differences in tested genotype distributions were found only in* IL18 *rs360719 compared with chronic tubulointerstitial nephritic patients. This difference probably arose from the fact that pathology of chronic infective tubulointerstitial nephritis might have been associated with this specific polymorphism.

## 6. Conclusions

In Polish HD patients, T-cell cytokine gene polymorphisms and vitamin D pathway gene polymorphisms are not associated with ESRD due to type 2 DM nephropathy.* IL18 *polymorphism is worthy to be further investigated in chronic infective tubulointerstitial nephritic patients as being possibly associated with this disease.

## Figures and Tables

**Table 1:** HRM and RFLP conditions for the identification of polymorphisms genotyped in the vitamin D pathway related genes.

Gene symbol	rs number	Alleles	Primers for PCR amplification (5′-3′)	Annealing temp. (°C)	PCR product length (bp)	HRM^a^ analysis	RFLP^b^ analysis	
						Melting temp. range (°C)	Restriction enzyme	Restriction fragment length (bp)
*GC *	rs7041	G/T	F: GGAGGTGAGTTTATGGAACAGC	66.3	493		HaeIII	T = 493
			R: GGCATTAAGCTGGTATGAGGTC					G = 414 + 79
	rs1155563	C/T	F: GGTTATTCTAAGACTGTGCTCTTGC	63.0	116	71–78		
			R: ATGTGTTCTCACTGTTCGACTCC					
	rs2298849	C/T	F: TCCACTGGCAAAACACATTAC	60.6	118	73–83		
			R: GGGACATCTGCATTTATCCTG					

*RXRA *	rs10881578	A/G	F: TCTTGAGCAATGCCAGCAG	60.6	75	80–90		
			R: CCACAGCTCACACATCCAATC					
	rs10776909	C/T	F: CAGCCTGTGGCCTGCTCA	60.6	95	82–92		
			R: AACCTCCGGCCCTTGGAG					
	rs749759	A/G	F: ATAGGGCTTGCCTGCCTAGA	62.6	382		BstXI	A = 382
			R: CTCCACCATAGCCCAAGTGA					G = 243 + 139

*VDR *	rs1544410	A/G	F: GGAGACACAGATAAGGAAATAC	60.6	248		FspI	A (B) = 248
			R: CCGCAAGAAACCTCAAATAACA					G (b) = 175 + 73
	rs2228570	C/T	F: GCACTGACTCTGGCTCTGAC	72.5	341		FokI	C (F) = 341
			R: ACCCTCCTGCTCCTGTGGCT					T (f) = 282 + 59

^a^HRM analysis: high resolution melt analysis.

^b^RFLP analysis: restriction fragment length polymorphism analysis.

**Table 2:** Characteristics of hemodialysis patients (*n* = 893).

Parameter	Type 2 DM nephropathy	Other causes of ESRD	*P* value
Demographic data	*n* = 366	*n* = 527	

Male sex, *n* (% of all)	201 (54.9)	307 (58.3)	0.337^b^
Age at RRT beginning, years	62.9 ± 14.1	57.2 ± 17.2	**<**0.0001^c^
RRT duration, years	3.29 (0.06–28.0)	4.42 (0.12–28.2)	**<**0.0001^c^
Death rate, cases per 100 patient-years	0.48	0.42	
Death rate, cases per 100 RRT-years	7.97	4.63	

Clinical data	*n* = 332	*n* = 527	

Coronary artery disease, *n* (% of all)	174 (52.4)	168 (31.9)	**<**0.0001^b^
Myocardial infarction, *n* (% of all)	98 (29.5)	101 (19.2)	0.009^b^
Parathyroidectomy, *n* (% of all)	2 (0.60)	21 (3.98)	0.0009^b^
Treatment with cinacalcet hydrochloride	24 (7.2)	98 (18.6)	**<**0.0001^b^

Laboratory data	*n* = 366	*n* = 527	

Anti-HBc positive, *n* (% of all)	95 (26.0)	126 (23.9)	0.528^b^
HBsAg positive, *n* (% of all anti-HBc positive)	7 (7.4)	11 (8.7)	0.807^b^
Anti-HCV positive, *n* (% of all)	26 (7.1)	57 (10.8)	0.062^b^
HCV RNA positive, *n* (% of all anti-HCV positive)	14 (53.8)	39 (68.4)	0.225^b^
Responders to hepatitis B vaccine, *n* (% of all)	202 (55.2)	315 (59.8)	0.191^b^
25(OH)D (ng/mL)^a^	13.3 ± 3.9	14.5 ± 5.6	0.182^a,d^
Total calcium (mg/dL)	8.83 ± 0.67	8.91 ± 0.82	0.264^d^
Phosphates (mg/dL)	5.03 ± 1.44	5.25 ± 1.49	0.054^d^
PTH (pg/mL)	296 (12.9–3,757)	463 (12.7–3,741)	**<**0.0001^c^
Total alkaline phosphatase (U/L)	98.2 (25.8–1,353)	97.1 (40.5–1,684)	0.528^c^

25(OH)D: 25-hydroxycholecalciferol, anti-HBc: antibodies to core antigen of hepatitis B virus, anti-HCV: antibodies to hepatitis C virus, HBsAg: surface antigen of hepatitis B virus, DM: diabetes mellitus, ESRD: end-stage renal disease, HCV RNA: ribonucleic acid of hepatitis C virus, PTH: parathyroid hormone, and RRT: renal replacement therapy.

A significant difference is indicated using bold font.

^a^ *n* = 66 for type 2 DM nephropathy; *n* = 96 for other renal diseases.

^b^Fisher's exact test.

^c^Mann-Whitney test.

^d^Unpaired *t*-test, Welch corrected.

**Table 3:** Characteristics of hemodialysis patients grouped by a cause of ESRD.

Parameter	Type 2 DM nephropathy (1)	Chronic glomerulonephritis (2)	Chronic tubulointerstitial nephritis (3)	Hypertensive nephropathy (4)	*P* value
Demographic data	*n* = 366	*n* = 178	*n* = 118	*n* = 231	

Male sex, *n* (% of all)	201 (54.9)	110 (61.8)	63 (53.4)	134 (58.0)	0.386^b^
Age at RRT beginning, years	62.9 ± 14.1	47.4 ± 17.6	59.9 ± 16.6	63.3 ± 13.6	**<**0.0001^c^ **1 versus 2**: **<**0.001^c^ **2 versus 3**: **<**0.001^c^ **2 versus 4**: **<**0.001^c^
RRT duration, years	3.29 (0.06–28.0)	5.73 (0.16–28.2)	4.82 (0.33–26.5)	3.82 (0.12–20.4)	**<**0.0001^c^ **1 versus 2**: **<**0.001^c^ **1 versus 3**: **<**0.01^c^ **2 versus 4**: **<**0.001^c^
Death rate, cases per 100 patient-years	0.48	0.41	0.44	0.42	
Death rate, cases per 100 dialysis-years	7.97	2.87	5.28	6.70	

Clinical data	*n* = 332	*n* = 178	*n* = 118	*n* = 231	

Coronary artery disease, *n* (% of all)	174 (52.4)	43 (24.2)	29 (24.6)	96 (41.5)	**<**0.0001^b^ **1 versus 2**: **<**0.0001^e^ **1 versus 3**: **<**0.0001^e^ **1 versus 4**: 0.013^e^ **2 versus 4**: 0.0002^e^ **3 versus 4**: 0.002^e^
Myocardial infarction, *n* (% of all)	98 (29.5)	25 (14.0)	17 (14.4)	59 (25.5)	**<**0.0001^b^ **1 versus 2**: **<**0.0001^e^ **1 versus 3**: **<**0.0001^e^ **1 versus 4**: **<**0.0001^e^ **2 versus 4**: 0.005^e^ **3 versus 4**: 0.02^e^
PTX, *n* (% of all)	2 (0.60)	14 (7.9)	5 (4.2)	2 (0.87)	**<**0.0001^b^ **1 versus 2**: **<**0.0001^e^ **1 versus 3**: 0.015^e^ **2 versus 4**: 0.0004^e^ **3 versus 4**: 0.046^e^
Treatment with cinacalcet hydrochloride	24 (7.2)	48 (27.0)	21 (17.8)	29 (12.6)	**<**0.0001^b^ **1 versus 2**: **<**0.0001^e^ **1 versus 3**: 0.0008^e^ **1 versus 4**: 0.017^e^ **2 versus 4**: 0.0003^e^

Laboratory data	*n* = 366	*n* = 178	*n* = 118	*n* = 231	

Anti-HBc positive, *n* (% of all)	95 (26.0)	53 (29.8)	25 (21.2)	48 (20.8)	0.233^b^
HBsAg positive, *n* (% of all anti-HBc positive)	7 (7.4)	10 (18.9)	0 (0.0)	1 (2.08)	0.0007^b^ **1 versus 2**: 0.032^e^ **2 versus 3**: 0.007^e^ **2 versus 4**: 0.001^e^
Anti-HCV positive, *n* (% of all)	26 (7.1)	33 (18.5)	11 (9.3)	13 (5.6)	**<**0.0001^b^ **1 versus 2**: 0.0004^e^ **2 versus 3**: 0.031^e^ **2 versus 4**: **<**0.0001^e^
HCV RNA positive, *n* (% of all anti-HCV positive)	14 (53.8)	27 (81.8)	4 (36.4)	8 (61.5)	**<**0.0001^b^ **1 versus 2**: **<**0.0001^e^ **2 versus 3**: 0.0004^e^ **2 versus 4**: **<**0.0001^e^
Responders to hepatitis B vaccine, *n* (% of all)	202 (55.2)	107 (60.1)	70 (59.3)	138 (59.7)	0.598^b^
25(OH)D (ng/mL)^a^	13.3 ± 3.9	14.2 ± 7.3	15.7 ± 4.3	14.1 ± 3.9	0.453^d^
Total calcium (mg/dL)	8.83 ± 0.67	8.85 ± 0.85	9.04 ± 0.61	8.88 ± 0.87	0.239^d^
Phosphates (mg/dL)	5.03 ± 1.44	5.63 ± 1.59	4.92 ± 1.29	5.15 ± 1.47	0.0007^d^ **1 versus 2**: **<**0.001^c^ **2 versus 3**: **<**0.01^c^ **2 versus 4**: **<**0.05^c^
PTH (pg/mL)	296 (12.9–3,757)	632 (12.7–3,118)	426 (45.8–3,741)	364 (19.5–2,351)	**<**0.0001^c^ **1 versus 2**: **<**0.001^c^ **1 versus 3**: **<**0.05^c^ **1 versus 4**: **<**0.05^c^ **2 versus 4**: **<**0.001^c^
Total ALP (U/L)	98.2 (25.8–1,353)	113 (44.5–860)	89.0 (40.5–1,684)	90.9 (41.0–1,110)	0.010^c^ **2 versus 4**: **<**0.05^c^

25(OH)D: 25-hydroxycholecalciferol, anti-HBc: antibodies to core antigen of hepatitis B virus, anti-HCV: antibodies to hepatitis C virus, HBsAg: surface antigen of hepatitis B virus, DM: diabetes mellitus, ESRD: end-stage renal disease, HCV RNA: ribonucleic acid of hepatitis C virus, PTH: parathyroid hormone, and RRT: renal replacement therapy.

^a^ *n* = 66 for type 2 DM nephropathy, *n* = 40 for chronic glomerulonephritis, *n* = 13 for chronic interstitial nephritis, and *n* = 43 for hypertensive nephropathy.

^b^Chi squared test.

^c^Kruskal-Wallis test.

^d^ANOVA test.

^e^Fisher's exact test.

**Table 4:** Comparison of the distribution of polymorphic variants of tested genes between ESRD patients treated with hemodialysis due to type 2 DM nephropathy and healthy subjects.

Parameter	Type 2 DM nephropathy (frequency)	Healthy subjects (frequency)	Odds ratio (95% CI)	Two-tailed *P*	*P*_trend_
*IL18* rs360719	*n* = 248	*n* = 240			

TT	133 (0.54)	121 (0.50)	Referent		0.233
CT	102 (0.41)	98 (0.41)	0.947 (0.654–1.372)	0.777	
CC	13 (0.05)	21 (0.09)	0.563 (0.270–1.174)	0.145	
CT + CC	115 (0.46)	119 (0.50)	0.879 (0.616–1.254)	0.526	
MAF	128 (0.26)	140 (0.29)	0.845 (0.638–1.119)	0.268	

*IL12A* rs568408	*n* = 234	*n* = 240			

GG	173 (0.74)	171 (0.71)	Referent		0.782
AG	52 (0.22)	63 (0.26)	0.816 (0.534–1.246)	0.389	
AA	9 (0.04)	6 (0.03)	1.483 (0.517–4.256)	0.600	
AG + AA	61 (0.26)	69 (0.29)	0.874 (0.583–1.309)	0.538	
MAF	70 (0.15)	75 (0.16)	0.976 (0.684–1.393)	0.965	

*IL12B* rs3212227	*n* = 247	*n* = 240			

AA	156 (0.63)	151 (0.63)	Referent		0.639
AC	84 (0.34)	77 (0.32)	1.056 (0.721–1.547)	0.846	
CC	7 (0.03)	12 (0.05)	0.563 (0.217–1.473)	0.345	
AC + CC	91 (0.37)	89 (0.37)	0.990 (0.685–1.430)	1.000	
MAF	98 (0.20)	101 (0.21)	0.927 (0.680–1.268)	0.699	

*IL4R *rs1805015	*n* = 303	*n* = 225			

TT	205 (0.68)	162 (0.72)	Referent		0.304
CT	82 (0.27)	53 (0.24)	1.223 (0.818–1.828)	0.360	
CC	16 (0.05)	10 (0.04)	1.264 (0.559–2.861)	0.684	
CT + CC	98 (0.32)	63 (0.28)	1.229 (0.843–1.793)	0.295	
MAF	114 (0.19)	73 (0.16)	1.197 (0.866–1.653)	0.313	

*IL13 *rs20541	*n* = 303	*n* = 230			

CC	168 (0.55)	124 (0.54)	Referent		0.457
CT	114 (0.38)	84 (0.36)	1.002 (0.695–1.443)	1.000	
TT	21 (0.07)	22 (0.10)	0.705 (0.371–1.338)	0.324	
CT + TT	135 (0.45)	106 (0.46)	0.940 (0.666–1.326)	0.726	
MAF	156 (0.26)	128 (0.28)	0.899 (0.684–1.182)	0.489	

*IL28B rs8099917 *	*n* = 339	*n* = 375			

TT	219 (0.65)	245 (0.65)	Referent		0.504
GT	107 (0.31)	123 (0.33)	0.973 (0.709–1.336)	0.872	
GG	13 (0.04)	7 (0.02)	2.078 (0.814–5.302)	0.169	
GT + GG	120 (0.35)	130 (0.35)	1.033 (0.759–1.405)	0.875	
MAF	133 (0.20)	137 (0.18)	1.092 (0.837–1.423)	0.560	

*IL28B rs12979860 *	*n* = 336	*n* = 372			

CC	141 (0.42)	164 (0.44)	Referent		0.669
CT	157 (0.47)	166 (0.45)	1.100 (0.804–1.505)	0.576	
TT	38 (0.11)	42 (0.11)	1.052 (0.643–1.723)	0.900	
CT + TT	195 (0.56)	208 (0.56)	1.090 (0.809–1.469)	0.595	
MAF	116 (0.29)	250 (0.34)	1.049 (0.842–1.307)	0.713	

*GC *rs2298849	*n* = 364^a^	*n* = 375			

TT	226 (0.62)	237 (0.63)	Referent		0.250
CT	110 (0.30)	124 (0.33)	0.930 (0.679–1.274)	0.688	
CC	28 (0.08)	14 (0.04)	2.097 (1.077–4.086)	0.035	
CT + CC	138 (0.38)	138 (0.37)	1.049 (0.778–1.413)	0.762	
MAF	166 (0.23)	152 (0.20)	1.162 (0.907–1.490)	0.262	

*GC *rs7041	*n* = 343	*n* = 361			

GG	112 (0.33)	116 (0.32)	Referent		0.572
GT	163 (0.47)	186 (0.52)	0.908 (0.650–1.268)	0.609	
TT	68 (0.20)	59 (0.16)	1.194 (0.773–1.844)	0.440	
GT + TT	231 (0.67)	245 (0.68)	0.977 (0.712–1.339)	0.936	
MAF	299 (0.44)	304 (0.42)	1.062 (0.860–1.312)	0.612	

*GC *rs1155563	*n* = 362	*n* = 377			

TT	180 (0.50)	189 (0.50)	Referent		0.541
CT	141 (0.39)	155 (0.41)	0.955 (0.703–1.297)	0.815	
CC	41 (0.11)	33 (0.09)	1.305 (0.789–2.155)	0.311	
CT + CC	182 (0.50)	188 (0.50)	1.017 (0.762–1.356)	0.941	
MAF	223 (0.31)	221 (0.29)	1.074 (0.859–1.341)	0.567	

*VDR *rs2228570	*n* = 345	*n* = 371			

CC	101 (0.29)	103 (0.28)	Referent		0.401
CT	175 (0.51)	183 (0.49)	0.975 (0.691–1.376)	0.930	
TT	69 (0.20)	85 (0.23)	0.828 (0.544–1.260)	0.394	
CT + TT	244 (0.71)	268 (0.72)	0.929 (0.671–1.285)	0.679	
MAF	313 (0.45)	353 (0.48)	0.915 (0.743–1.126)	0.432	

*VDR *rs1544410	*n* = 359	*n* = 372			

GG	137 (0.38)	148 (0.40)	Referent		0.753
AG	165 (0.46)	165 (0.44)	1.080 (0.787–1.483)	0.686	
AA	57 (0.16)	59 (0.16)	1.044 (0.678–1.607)	0.912	
AG + AA	222 (0.62)	224 (0.60)	1.071 (0.795–1.442)	0.705	
MAF	279 (0.39)	283 (0.38)	1.035 (0.839–1.278)	0.788	

*RXRA *rs10776909	*n* = 364	*n* = 378			

CC	233 (0.64)	250 (0.66)	Referent		0.426
CT	111 (0.30)	112 (0.30)	1.063 (0.774–1.461)	0.746	
TT	20 (0.05)	16 (0.04)	1.341 (0.679–2.651)	0.490	
CT + TT	131 (0.36)	128 (0.34)	1.098 (0.812–1.485)	0.590	
MAF	151 (0.21)	144 (0.19)	1.112 (0.862–1.435)	0.452	

*RXRA *rs10881578	*n* = 365	*n* = 377			

AA	197 (0.54)	183 (0.48)	Referent		0.168
AG	134 (0.37)	154 (0.41)	0.808 (0.775–1.046)	0.185	
GG	34 (0.09)	40 (0.11)	0.790 (0.479–1.301)	0.376	
AG + GG	168 (0.46)	194 (0.51)	0.804 (0.603–1.073)	0.143	
MAF	202 (0.28)	234 (0.31)	0.850 (0.680–1.063)	0.172	

*RXRA *rs749759	*n* = 355	*n* = 370			

GG	207 (0.58)	221 (0.60)	Referent		0.850
AG	125 (0.35)	123 (0.33)	1.085 (0.794–1.216)	0.632	
AA	23 (0.06)	26 (0.07)	0.944 (0.522–1.708)	0.881	
AG + AA	148 (0.42)	149 (0.40)	1.061 (0.789–1.426)	0.706	
MAF	171 (0.24)	175 (0.24)	1.024 (0.804–1.304)	0.894	

ESRD: end-stage renal disease, DM: diabetes mellitus, and MAF: minor allele frequency.

^a^Not consistent with Hardy-Weinberg equilibrium.

**Table 5:** Comparison of the distribution of polymorphic variants of tested genes between ESRD patients treated with hemodialysis due to type 2 DM nephropathy and the most common causes of ESRD other than type 2 DM nephropathy (chronic glomerulonephritis, chronic tubulointerstitial nephritis, and hypertensive nephritis).

Genotype	Type 2 DM nephropathy (frequency)	Other causes of ESRD (frequency)	Odds ratio (95% CI)	Two-tailed *P*	*P*_trend_
*IL18* rs360719	*n* = 248	*n* = 353			

TT	133 (0.54)	186 (0.53)	Referent	—	0.362
CT	102 (0.41)	135 (0.38)	1.057 (0.752–1.485)	0.795	
CC	13 (0.05)	32 (0.09)	0.568 (0.287–1.124)	0.107	
CT + CC	115 (0.46)	167 (0.47)	0.963 (0.696–1.334)	0.868	
MAF	128 (0.26)	199 (0.28)	0.886 (0.684–1.149)	0.370	

*IL12A* rs568408	*n* = 234	*n* = 337			

GG	173 (0.74)	234 (0.69)	Referent	—	0.303
AG	52 (0.22)	89 (0.26)	0.790 (0.533–1.060)	0.275	
AA	9 (0.04)	14 (0.04)	0.870 (0.368–2.055)	0.831	
AG + AA	61 (0.26)	103 (0.31)	0.801 (0.552–1.163)	0.260	
MAF	70 (0.15)	117 (0.17)	0.837 (0.606–1.157)	0.319	

*IL12B* rs3212227	*n* = 247	*n* = 352			

AA	156 (0.63)	205 (0.58)	Referent	—	0.176
AC	84 (0.34)	132 (0.38)	0.836 (0.593–1.068)	0.337	
CC	7 (0.03)	15 (0.04)	0.613 (0.244–1.540)	0.376	
AC + CC	91 (0.37)	147 (0.42)	0.814 (0.582–1.136)	0.236	
MAF	98 (0.20)	162 (0.23)	0.828 (0.624–1.098)	0.215	

*IL4R *rs1805015	*n* = 303	*n* = 436			

TT	205 (0.68)	295 (0.68)	Referent	—	0.871
CT	82 (0.27)	121 (0.28)	0.975 (0.700–2.360)	0.933	
CC	16 (0.05)	20 (0.05)	1.151 (0.583–2.275)	0.728	
CT + CC	98 (0.32)	141 (0.32)	1.000 (0.731–1.368)	1.000	
MAF	114 (0.19)	161 (0.18)	1.023 (0.784–1.335)	0.919	

*IL13 *rs20541	*n* = 303	*n* = 436			

CC	168 (0.55)	242 (0.56)	Referent	—	0.902
CT	114 (0.38)	166 (0.38)	0.989 (0.726–1.348)	1.000	
TT	21 (0.07)	28 (0.06)	1.080 (0.594–1.967)	0.878	
CT + TT	135 (0.45)	194 (0.44)	1.002 (0.746–1.346)	1.000	
MAF	156 (0.26)	222 (0.25)	1.015 (0.800–1.287)	0.950	

*IL28B rs8099917 *	*n* = 339	*n* = 493			

TT	219 (0.65)	317 (0.64)	Referent	—	0.858
GT	107 (0.31)	162 (0.33)	0.956 (0.709–1.289)	0.820	
GG	13 (0.04)	14 (0.03)	1.344 (0.620–2.916)	0.549	
GT + GG	120 (0.35)	176 (0.36)	0.987 (0.739–1.318)	0.941	
MAF	133 (0.20)	190 (0.19)	1.022 (0.799–1.309)	0.910	

*IL28B *rs12979860	*n* = 336	*n* = 488			

CC	141 (0.42)	209 (0.43)	Referent	—	0.952
CT	157 (0.47)	221 (0.45)	1.053 (0.783–1.415)	0.763	
TT	38 (0.11)	58 (0.12)	0.971 (0.612–1.541)	0.907	
CT + TT	195 (0.56)	279 (0.57)	1.036 (0.782–1.373)	0.830	
MAF	116 (0.29)	337 (0.35)	1.006 (0.819–1.237)	0.994	

*GC *rs2298849	*n* = 364^a^	*n* = 524			

TT	226 (0.62)	339 (0.65)	Referent	—	0.109
CT	110 (0.30)	165 (0.31)	1.000 (0.745–1.342)	1.000	
CC	28 (0.08)	20 (0.04)	2.100 (1.155–3.819)	0.014	
CT + CC	138 (0.38)	185 (0.35)	1.119 (0.848–1.477)	0.436	
MAF	166 (0.23)	205 (0.20)	1.215 (0.964–1.530)	0.111	

*GC *rs7041	*n* = 343	*n* = 506			

GG	112 (0.33)	182 (0.36)	Referent	—	0.247
GT	163 (0.47)	236 (0.47)	1.122 (0.824–1.528)	0.480	
TT	68 (0.20)	88 (0.17)	1.256 (0.846–1.863)	0.267	
GT + TT	231 (0.67)	324 (0.64)	1.159 (0.867–1.548)	0.340	
MAF	299 (0.44)	412 (0.41)	1.125 (0.925–1.369)	0.259	

*GC *rs1155563	*n* = 362	*n* = 527			

TT	180 (0.50)	252 (0.48)	Referent	—	0.614
CT	141 (0.39)	213 (0.40)	0.927 (0.696–1.234)	0.610	
CC	41 (0.11)	62 (0.12)	0.926 (0.597–1.435)	0.740	
CT + CC	182 (0.50)	275 (0.52)	0.927 (0.709–1.211)	0.585	
MAF	223 (0.31)	337 (0.32)	0.947 (0.772–1.161)	0.638	

*VDR *rs2228570	*n* = 345	*n* = 503			

CC	101 (0.29)	130 (0.26)	Referent	—	0.541
CT	175 (0.51)	275 (0.55)	0.819 (0.594–1.130)	0.249	
TT	69 (0.20)	98 (0.19)	0.906 (0.606–1.356)	0.682	
CT + TT	244 (0.71)	373 (0.74)	0.842 (0.620–1.143)	0.273	
MAF	313 (0.45)	471 (0.47)	0.943 (0.776–1.145)	0.588	

*VDR *rs1544410	*n* = 359	*n* = 512			

GG	137 (0.38)	189 (0.37)	Referent	—	0.598
AG	165 (0.46)	235 (0.46)	0.969 (0.720–1.303)	0.880	
AA	57 (0.16)	88 (0.17)	0.894 (0.599–1.332)	0.613	
AG + AA	222 (0.62)	323 (0.63)	0.948 (0.718–1.253)	0.722	
MAF	279 (0.39)	411 (0.40)	0.948 (0.778–1.152)	0.626	

*RXRA *rs10776909	*n* = 364	*n* = 526			

CC	233 (0.64)	308 (0.59)	Referent	—	0.298
CT	111 (0.30)	196 (0.37)	0.749 (0.561–0.999)	0.050	
TT	20 (0.05)	22 (0.04)	1.202 (0.641–2.254)	0.629	
CT + TT	131 (0.36)	218 (0.41)	0.794 (0.603–1.046)	0.108	
MAF	151 (0.21)	240 (0.23)	0.883 (0.702–1.112)	0.317	

*RXRA rs10881578 *	*n* = 365	*n* = 525			

AA	197 (0.54)	252 (0.48)	Referent	—	0.134
AG	134 (0.37)	220 (0.42)	0.779 (0.586–1.035)	0.096	
GG	34 (0.09)	53 (0.10)	0.821 (0.513–1.312)	0.478	
AG + GG	168 (0.46)	273 (0.52)	0.787 (0.602–1.029)	0.088	
MAF	202 (0.28)	326 (0.31)	0.850 (0.690–1.046)	0.139	

*RXRA *rs749759	*n* = 355	*n* = 514			

GG	207 (0.58)	265 (0.52)	Referent	—	0.082
AG	125 (0.35)	212 (0.41)	0.755 (0.567–1.005)	0.059	
AA	23 (0.06)	37 (0.07)	0.796 (0.459–1.381)	0.490	
AG + AA	148 (0.42)	249 (0.48)	0.761 (0.579–1.000)	0.053	
MAF	171 (0.24)	286 (0.28)	0.823 (0.661–1.025)	0.092	

ESRD: end-stage renal disease, DM: diabetes mellitus, and MAF: minor allele frequency.

^a^Not consistent with Hardy-Weinberg equilibrium.

**Table 6:** Selected comparisons of the polymorphic variants distribution of tested genes between type 2 DM nephropathy patients, chronic infective tubulointerstitial nephritic patients, and healthy subjects.

Genotype	Genotype frequencies		Odds ratio (95% CI)	Two-tailed *P*	*P*_trend_
Type 2 DM nephropathy versus chronic infective tubulointerstitial nephritis					
*IL18* rs360719	*n* = 248	*n* = 77			

TT	133 (0.54)	54 (0.70)	Referent		**0.033**
CT	102 (0.41)	19 (0.25)	2.180 (1.217–3.905)	0.009^a^	
CC	13 (0.05)	4 (0.05)	1.320 (0.412–4.228)	0.783	
CT + CC	115 (0.46)	23 (0.30)	2.030 (1.173–3.512)	0.012^a^	
MAF	128 (0.26)	27 (0.18)	1.636 (1.031–2.596)	**0.046**	

Chronic infective tubulointerstitial nephritis versus healthy controls					
*IL18* rs360719	*n* = 77	*n* = 240			

TT	54 (0.70)	121 (0.50)	Referent		**0.005**
CT	19 (0.25)	98 (0.41)	0.434 (0.242–0.781)	0.006^a^	
CC	4 (0.05)	21 (0.09)	0.427 (0.140–1.303)	0.160	
CT + CC	23 (0.30)	119 (0.50)	0.433 (0.250–0.750)	0.004^a^	
MAF	27 (0.18)	140 (0.29)	0.516 (0.326–0.818)	**0.006**	

DM: diabetes mellitus; MAF: minor allele frequency.

Significant differences are indicated using bold font.

^a^Significant after the Bonferroni correction (*P* < 0.017).

**Table 7:** Comparison of the distribution of polymorphic variants of tested genes between ESRD patients treated with hemodialysis due to type 2 DM nephropathy grouped by diagnosis of CAD.

Parameter	Type 2 DM nephropathy with CAD (frequency)	Type 2 DM nephropathy without CAD (frequency)	Odds ratio (95% CI)	Two-tailed *P*	*P*_trend_
*IL18* rs360719	*n* = 124	*n* = 109			

TT	68 (0.55)	53 (0.49)	Referent		0.269
CT	51 (0.41)	49 (0.45)	1.128 (0.725–1.754)	0.653	
CC	5 (0.04)	7 (0.06)	0.628 (0.194–2.036)	0.557	
CT + CC	56 (0.45)	56 (0.51)	0.879 (0.560–1.380)	0.645	
MAF	61 (0.25)	63 (0.29)	0.803 (0.532–1.211)	0.345	

*IL12A* rs568408	*n* = 117	*n* = 102			

GG	83 (0.71)	77 (0.63)	Referent		0.361
AG	28 (0.24)	22 (0.22)	1.181 (0.623–2.236)	0.630	
AA	6 (0.05)	3 (0.03)	1.855 (0.448–7.678)	0.502	
AG + AA	34 (0.29)	25 (0.25)	1.262 (0.691–2.304)	0.542	
MAF	40 (0.17)	28 (0.14)	1.311 (0.776–2.214)	0.378	

*IL12B* rs3212227	*n* = 124	*n* = 109			

AA	78 (0.63)	69 (0.63)	Referent		0.906
AC	43 (0.35)	36 (0.33)	1.057 (0.611–1.829)	0.889	
CC	3 (0.02)	4 (0.04)	0.664 (0.143–3.069)	0.708	
AC + CC	46 (0.37)	40 (0.37)	1.017 (0.597–1.734)	1.000	
MAF	49 (0.20)	44 (0.20)	0.974 (0.618–1.535)	0.909	

*IL4R *rs1805015	*n* = 144	*n* = 127			

TT	95 (0.66)	86 (0.68)	Referent		0.947
CT	42 (0.29)	32 (0.25)	1.188 (0.689–2.048)	0.581	
CC	7 (0.05)	9 (0.07)	0.704 (0.251–1.972)	0.605	
CT + CC	49 (0.34)	41 (0.32)	1.082 (0.652–1.797)	0.797	
MAF	56 (0.19)	50 (0.20)	0.985 (0.644–1.504)	0.944	

*IL13 *rs20541	*n* = 144	*n* = 127			

CC	80 (0.56)	71 (0.56)	Referent		0.867
CT	55 (0.38)	46 (0.36)	1.061 (0.640–1.759)	0.898	
TT	9 (0.06)	10 (0.08)	0.799 (0.307–2.077)	0.808	
CT + TT	64 (0.44)	56 (0.44)	1.014 (0.627–1.640)	1.000	
MAF	73 (0.25)	92 (0.26)	0.967 (0.657–1.423)	0.944	

*IL28B rs8099917 *	*n* = 163	*n* = 145			

TT	105 (0.64)	97 (0.67)	Referent		0.752
GT	52 (0.32)	42 (0.29)	1.144 (0.700–1.870)	0.618	
GG	6 (0.04)	6 (0.04)	0.924 (0.288–2.961)	1.000	
GT + GG	58 (0.36)	48 (0.33)	1.116 (0.697–1.189)	0.719	
MAF	64 (0.20)	54 (0.19)	1.068 (0.714–1.597)	0.829	

*IL28B rs12979860 *	*n* = 163	*n* = 142			

CC	69 (0.42)	66 (0.46)	Referent		0.352
CT	73 (0.45)	62 (0.44)	1.126 (0.698–1.816)	0.715	
TT	21 (0.13)	14 (0.10)	1.435 (0.674–3.055)	0.448	
CT + TT	94 (0.58)	76 (0.54)	1.183 (0.752–1.861)	0.490	
MAF	115 (0.35)	90 (0.32)	1.175 (0.838–1.647)	0.396	

*GC rs2298849 *	*n* = 172	*n* = 158^a^			

TT	99 (0.58)	106 (0.67)	Referent		0.173
CT	60 (0.35)	40 (0.25)	1.606 (0.989–2.608)	0.067	
CC	13 (0.07)	12 (0.08)	1.160 (0.505–2.663)	0.833	
CT + CC	73 (0.42)	52 (0.33)	1.503 (0.959–2.355)	0.088	
MAF	166 (0.25)	64 (0.20)	1.313 (0.909–1.895)	0.174	

*GC rs7041 *	*n* = 161	*n* = 151			

GG	57 (0.35)	46 (0.30)	Referent		0.844
GT	69 (0.43)	82 (0.54)	1.327 (0.825–2.134)	0.277	
TT	35 (0.22)	23 (0.15)	1.629 (0.900–2.949)	0.136	
GT + TT	104 (0.65)	105 (0.70)	1.061 (0.721–1.559)	0.769	
MAF	139 (0.43)	128 (0.42)	1.025 (0.746–1.409)	0.943	

*GC rs1155563 *	*n* = 172	*n* = 157			

TT	82 (0.48)	79 (0.50)	Referent		0.645
CT	70 (0.41)	61 (0.39)	1.106 (0.697–1.755)	0.724	
CC	20 (0.12)	17 (0.11)	1.133 (0.554–2.321)	0.856	
CT + CC	90 (0.52)	78 (0.50)	1.112 (0.721–1.714)	0.660	
MAF	110 (0.32)	95 (0.30)	1.084 (0.779–1.508)	0.695	

*VDR *rs2228570	*n* = 162	*n* = 152			

CC	43 (0.27)	44 (0.29)	Referent		0.316
CT	93 (0.57)	68 (0.45)	1.400 (0.829–2.363)	0.230	
TT	26 (0.16)	40 (0.26)	0.665 (0.348–1.272)	0.252	
CT + TT	119 (0.73)	108 (0.71)	1.128 (0.688–1.849)	0.705	
MAF	145 (0.45)	148 (0.49)	0.854 (0.624–1.169)	0.365	

*VDR *rs1544410	*n* = 170	*n* = 155			

GG	65 (0.38)	61 (0.39)	Referent		0.772
AG	79 (0.46)	72 (0.46)	1.030 (0.641–1.653)	0.905	
AA	26 (0.15)	22 (0.14)	1.109 (0.569–2.160)	0.865	
AG + AA	105 (0.62)	94 (0.61)	1.048 (0.671–1.639)	0.909	
MAF	131 (0.39)	116 (0.37)	1.048 (0.763–1.440)	0.833	

*RXRA *rs10776909	*n* = 172	*n* = 158			

CC	112 (0.65)	104 (0.66)	Referent		0.621
CT	48 (0.28)	47 (0.30)	0.948 (0.585–1.537)	0.902	
TT	12 (0.07)	7 (0.04)	1.592 (0.604–4.198)	0.473	
CT + TT	60 (0.35)	54 (0.34)	1.032 (0.655–1.625)	0.908	
MAF	72 (0.21)	61 (0.19)	1.107 (0.756–1.621)	0.672	

*RXRA rs10881578 *	*n* = 173	*n* = 158			

AA	89 (0.51)	92 (0.58)	Referent		0.192
AG	65 (0.38)	53 (0.34)	1.268 (0.796–2.019)	0.345	
GG	19 (0.11)	13 (0.08)	1.511 (0.704–3.241)	0.340	
AG + GG	84 (0.49)	66 (0.42)	1.316 (0.852–2.032)	0.226	
MAF	103 (0.30)	79 (0.25)	1.272 (0.902–1.793)	0.199	

*RXRA rs749759 *	*n* = 169	*n* = 153			

GG	100 (0.59)	89 (0.58)	Referent		0.812
AG	59 (0.35)	54 (0.35)	0.972 (0.610–1.551)	1.000	
AA	10 (0.06)	10 (0.07)	0.890 (0.354–2.238)	0.818	
AG + AA	69 (0.41)	64 (0.42)	0.960 (0.615–1.496)	0.910	
MAF	79 (0.23)	74 (0.24)	0.956 (0.665–1.375)	0.882	

CAD: coronary artery disease, ESRD: end-stage renal disease, DM: diabetes mellitus, and MAF: minor allele frequency.

^a^Not consistent with Hardy-Weinberg equilibrium.

**Table 8:** Comparison of the distribution of polymorphic variants of tested genes between type 2 DM nephropathy patients with diagnosis of CAD and healthy controls.

Parameter	Type 2 DM nephropathy with CAD (frequency)	Healthy controls (frequency)	Odds ratio (95% CI)	Two-tailed *P*	*P*_trend_
*IL18* rs360719	*n* = 124	*n* = 240			

TT	68 (0.55)	121 (0.50)	Referent		0.186
CT	51 (0.41)	98 (0.41)	0.926 (0.590–1.453)	0.819	
CC	5 (0.04)	21 (0.09)	0.424 (0.153–1.174)	0.122	
CT + CC	56 (0.45)	119 (0.50)	0.837 (0.542–1.294)	0.440	
MAF	61 (0.25)	140 (0.29)	0.792 (0.558–1.124)	0.223	

*IL12A* rs568408	*n* = 117	*n* = 240			

GG	83 (0.71)	171 (0.71)	Referent		0.626
AG	28 (0.24)	63 (0.26)	0.916 (0.546–1.535)	0.794	
AA	6 (0.05)	6 (0.03)	2.060 (0.645–6.583)	0.348	
AG + AA	34 (0.29)	69 (0.29)	1.015 (0.624–1.653)	1.000	
MAF	40 (0.17)	75 (0.16)	1.113 (0.731–1.695)	0.695	

*IL12B* rs3212227	*n* = 124	*n* = 240			

AA	78 (0.63)	151 (0.63)	Referent		0.475
AC	43 (0.35)	77 (0.32)	1.081 (0.681–1.717)	0.813	
CC	3 (0.02)	12 (0.05)	0.484 (0.133–1.766)	0.397	
AC + CC	46 (0.37)	89 (0.37)	1.001 (0.639–1.567)	1.000	
MAF	49 (0.20)	101 (0.21)	0.924 (0.631–1.354)	0.757	

*IL4R *rs1805015	*n* = 144	*n* = 225			

TT	95 (0.66)	162 (0.72)	Referent		0.285
CT	42 (0.29)	53 (0.24)	1.351 (0.838–2.179)	0.221	
CC	7 (0.05)	10 (0.04)	1.194 (0.440–3.240)	0.798	
CT + CC	49 (0.34)	63 (0.28)	1.326 (0.845–2.083)	0.246	
MAF	56 (0.19)	73 (0.16)	1.247 (0.848–1.832)	0.305	

*IL13 *rs20541	*n* = 144	*n* = 230			

CC	80 (0.56)	124 (0.54)	Referent		0.469
CT	55 (0.38)	84 (0.36)	1.015 (0.653–1.578)	1.000	
TT	9 (0.06)	22 (0.10)	0.634 (0.278–1.447)	0.324	
CT + TT	64 (0.44)	106 (0.46)	0.936 (0.616–1.422)	0.831	
MAF	73 (0.25)	128 (0.28)	0.881 (0.630–1.231)	0.510	

*IL28B rs8099917 *	*n* = 163	*n* = 375			

TT	105 (0.64)	245 (0.65)	Referent		0.584
GT	52 (0.32)	123 (0.33)	0.986 (0.663–1.467)	1.000	
GG	6 (0.04)	7 (0.02)	2.000 (0.656–6.094)	0.229	
GT + GG	58 (0.36)	130 (0.35)	1.041 (0.709–1.530)	0.845	
MAF	64 (0.20)	137 (0.18)	1.093 (0.786–1.521)	0.658	

*IL28B rs12979860 *	*n* = 163	*n* = 372			

CC	69 (0.42)	164 (0.44)	Referent		0.281
CT	73 (0.45)	166 (0.45)	1.045 (0.705–1.549)	0.841	
TT	21 (0.13)	42 (0.11)	1.188 (0.656–2.154)	0.644	
CT + TT	94 (0.58)	208 (0.56)	1.074 (0.740–1.558)	0.776	
MAF	115 (0.35)	250 (0.34)	1.077 (0.819–1.416)	0.644	

*GC rs2298849 *	*n* = 172	*n* = 375			

TT	99 (0.58)	237 (0.63)	Referent		0.080
CT	60 (0.35)	124 (0.33)	1.158 (0.786–1.706)	0.486	
CC	13 (0.07)	14 (0.04)	2.223 (1.008–4.901)	0.052	
CT + CC	73 (0.42)	138 (0.37)	1.266 (0.876–1.830)	0.220	
MAF	166 (0.25)	152 (0.20)	1.311 (0.969–1.774)	0.092	

*GC rs7041 *	*n* = 161	*n* = 361			

GG	57 (0.35)	116 (0.32)	Referent		0.748
GT	69 (0.43)	186 (0.52)	0.755 (0.496–1.150)	0.196	
TT	35 (0.22)	59 (0.16)	1.207 (0.714–2.040)	0.502	
GT + TT	104 (0.65)	245 (0.68)	0.864 (0.584–1.278)	0.482	
MAF	139 (0.43)	304 (0.42)	1.044 (0.801–1.362)	0.800	

*GC rs1155563 *	*n* = 172	*n* = 377			

TT	82 (0.48)	189 (0.50)	Referent		0.378
CT	70 (0.41)	155 (0.41)	1.041 (0.710–1.527)	0.845	
CC	20 (0.12)	33 (0.09)	1.397 (0.757–2.578)	0.332	
CT + CC	90 (0.52)	188 (0.50)	1.103 (0.769–1.583)	0.646	
MAF	110 (0.32)	221 (0.29)	1.134 (0.861–1.494)	0.411	

*VDR *rs2228570	*n* = 162	*n* = 371			

CC	43 (0.27)	103 (0.28)	Referent		0.386
CT	93 (0.57)	183 (0.49)	1.217 (0.788–1.880)	0.384	
TT	26 (0.16)	85 (0.23)	0.733 (0.416–1.290)	0.321	
CT + TT	119 (0.73)	268 (0.72)	1.064 (0.702–1.613)	0.833	
MAF	145 (0.45)	353 (0.48)	0.893 (0.687–1.160)	0.434	

*VDR *rs1544410	*n* = 170	*n* = 372			

GG	65 (0.38)	148 (0.40)	Referent		0.880
AG	79 (0.46)	165 (0.44)	1.090 (0.734–1.620)	0.687	
AA	26 (0.15)	59 (0.16)	1.003 (0.581–1.732)	1.000	
AG + AA	105 (0.62)	224 (0.60)	1.067 (0.735–1.549)	0.776	
MAF	131 (0.39)	283 (0.38)	1.021 (0.784–1.329)	0.931	

*RXRA *rs10776909	*n* = 172	*n* = 378			

CC	112 (0.65)	250 (0.66)	Referent		0.483
CT	48 (0.28)	112 (0.30)	0.957 (0.638–1.434)	0.838	
TT	12 (0.07)	16 (0.04)	1.674 (0.767–3.656)	0.209	
CT + TT	60 (0.35)	128 (0.34)	1.046 (0.716–1.529)	0.846	
MAF	72 (0.21)	144 (0.19)	1.125 (0.819–1.545)	0.518	

*RXRA rs10881578 *	*n* = 173	*n* = 377			

AA	89 (0.51)	183 (0.48)	Referent		0.682
AG	65 (0.38)	154 (0.41)	0.868 (0.591–1.275)	0.494	
GG	19 (0.11)	40 (0.11)	0.977 (0.535–1.783)	1.000	
AG + GG	84 (0.49)	194 (0.51)	0.890 (0.621–1.276)	0.582	
MAF	103 (0.30)	234 (0.31)	0.942 (0.714–1.243)	0.725	

*RXRA rs749759 *	*n* = 169	*n* = 370			

GG	100 (0.59)	221 (0.60)	Referent		0.924
AG	59 (0.35)	123 (0.33)	1.060 (0.718–1.566)	0.842	
AA	10 (0.06)	26 (0.07)	0.850 (0.395–1.830)	0.710	
AG + AA	69 (0.41)	149 (0.40)	1.023 (0.707–1.482)	0.925	
MAF	79 (0.23)	175 (0.24)	0.985 (0.727–1.334)	0.983	

CAD: coronary artery disease, DM: diabetes mellitus, and MAF: minor allele frequency.
